# Chronic kidney disease, proteinuria, and mortality risk in patients with Parkinson’s disease: a 12-year longitudinal study

**DOI:** 10.3389/fnagi.2025.1631079

**Published:** 2025-10-27

**Authors:** Kyoungwon Baik, Minwoong Kang, Yu Jeong Park, Su Jin Chung, Kyungmi Oh, Sung Hoon Kang, Seong-Beom Koh

**Affiliations:** ^1^Department of Neurology, Korea University Anam Hospital, Korea University College of Medicine, Seoul, Republic of Korea; ^2^Geriatric Health Clinic and Research Institute, Korea University College of Medicine, Seoul, Republic of Korea; ^3^Department of Biomedical Research Center, Korea University Guro Hospital, Korea University College of Medicine, Seoul, Republic of Korea; ^4^Department of Neurology, Korea University Guro Hospital, Korea University College of Medicine, Seoul, Republic of Korea; ^5^Department of Neurology, Inje University Ilsan Paik Hospital, Inje University College of Medicine, Goyang, Republic of Korea

**Keywords:** Parkinson’s disease, mortality, proteinuria, chronic kidney disease, kidney function

## Abstract

**Background:**

Various comorbidities contribute to mortality in patients with Parkinson’s disease (PD). Although growing evidence demonstrates that chronic kidney disease (CKD) increases the risk of developing PD, the effect of CKD on all-cause mortality remains unclear.

**Methods:**

We enrolled 59,293 patients aged ≥40 years with *de novo* PD between 2009 and 2015, using de-identified data from the Korean National Health Insurance Service. Cox proportional hazards regression analysis using the presence of CKD or proteinuria as a predictor was performed to investigate the association between CKD, proteinuria, and mortality. For sensitivity analysis, the degree of eGFR or proteinuria were used as predictors in place of CKD/proteinuria.

**Results:**

Parkinson’s disease patients with CKD (hazard ratio [HR] = 1.240, 95% confidence interval [CI] 1.190–1.283) and proteinuria (HR = 1.543, 95% CI 1.457–1.634) had a higher risk of mortality, even after controlling for confounding factors. The degree of kidney dysfunction (*p* < 0.001) and proteinuria (*p* < 0.001) were associated with an increased HR for mortality. Furthermore, female patients with CKD were more vulnerable to mortality than male patients (*p* for sex × CKD < 0.001); however, there was no sex-specific vulnerability of proteinuria to mortality (*p* for sex × proteinuria = 0.603).

**Conclusion:**

Chronic kidney disease and proteinuria were associated with a higher all-cause mortality in patients with PD in a dose-dependent manner. Furthermore, these results highlight that strategies for controlling kidney function are necessary to reduce mortality in patients with PD.

## 1 Introduction

Parkinson’s disease (PD), characterized by the progressive impairment of motor ability and a range of non-motor symptoms, including dementia, hallucinations, autonomic dysfunction, and mood disorders, is the second most common neurodegenerative disease worldwide. The associated motor and non-motor dysfunctions significantly disrupt patients’ daily lives, and PD is associated with higher mortality rates than in the general healthy population. Patients with PD face a 1.5-to 5-fold increased risk of mortality compared to those without this disease (de Lau and Breteler, 2006; Posada et al., 2011; Macleod et al., 2014; Ryu et al., 2023).

In addition to the disease’s inherent causes (Bäckström et al., 2018), such as cognitive impairment, freezing of gait, a postural imbalance and gait disorder phenotype, and disease severity, various comorbidities also contribute to the increased mortality in PD. Common comorbidities, such as cardiovascular disease, diabetes, and respiratory conditions, have been recognized for their impact on the survival of patients with PD (Posada et al., 2011; Macleod et al., 2016; Guilherme et al., 2021; Ke et al., 2024). However, the impact of chronic kidney disease (CKD), one of the leading causes of death globally, on the life span and survival of patients with PD remains unclear.

Prior research has presented evidence to indicate a potential link between CKD and the development of PD, although the specific mechanisms remain poorly understood. Epidemiological studies have shown an increased risk of PD in patients with CKD (Lin et al., 2012; Wang et al., 2014; Nam et al., 2019); suggesting that metabolic disturbances, hypoxia, uremia, vasogenic edema, and oxidative stress may play roles in PD development (Hamed, 2019; Meléndez-Flores and Estrada-Bellmann, 2021). However, the effect of CKD on all-cause mortality in patients undergoing PD remains unclear. In this study, we investigated whether CKD and proteinuria are associated with mortality rates in patients with PD, using data from a large nationwide cohort in Korea.

## 2 Materials and methods

### 2.1 Data source

This study used a customized dataset from the Korean National Health Insurance Service (KNHIS), which encompasses approximately 50 million individuals, representing more than 99% of the Korean population^1^. The KNHIS database includes personal information; health insurance claim codes (procedures and prescriptions); diagnostic codes from the Korean Standard Classification of Diseases, 7th Revision based on the International Classification of Diseases, 10th Revision (ICD-10); death records from the Korean National Statistical Office; and general health screening examination data for each participant from 2002 to 2020.

### 2.2 Study participants

**FIGURE 1:**
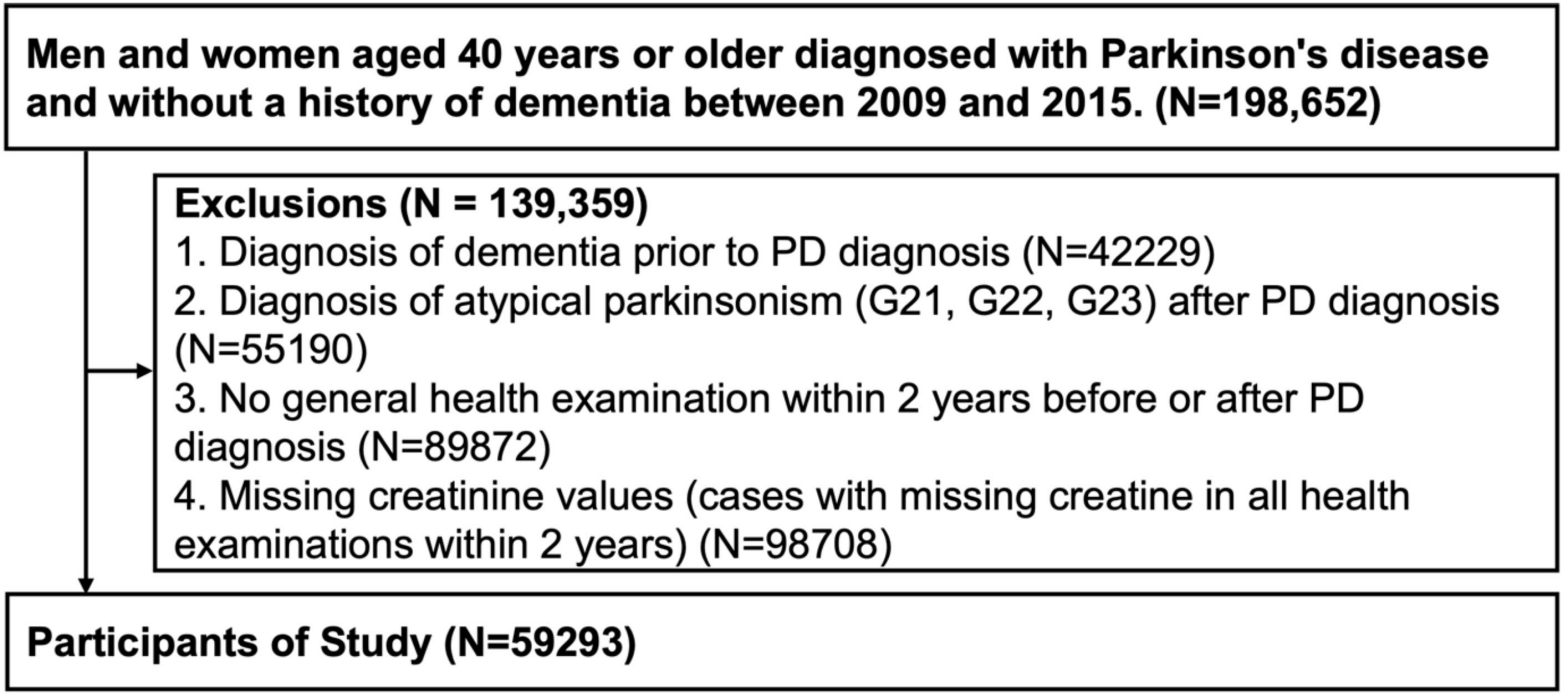
Flow chart of the study.

Patients aged ≥40 years diagnosed with PD between 2009 and 2015 were enrolled based on the ICD-10 codes (G20) and prescription of PD medication. In total, 198,652 eligible candidates were identified. Patients with the following conditions were excluded: 42,229 patients with a prior history of dementia, 55,190 patients diagnosed with an atypical Parkinsonism (G21, G22, G23) before or after PD diagnosis, 89,872 patients who did not undergo a general health examination within 2 years before or after their PD diagnosis, and 98,708 patients with missing creatinine values (Figure 1). This study was reviewed and approved by the Institutional Review Board of Korea University Guro Hospital. Due to the retrospective nature of the study, Institutional Review Board of Korea University Guro Hospital waived the need of obtaining informed consent. All procedures performed in human studies were in accordance with the ethical standards of the institutional and/or national research committee and with the 1964 Helsinki Declaration and its later amendments or comparable ethical standards.

### 2.3 Definition of chronic kidney disease

Patients with PD were divided into two groups: non-CKD and CKD. CKD was defined as an estimated glomerular filtration rate (eGFR) of <60, as calculated using the Chronic Kidney Disease Epidemiology Collaboration (CKD-EPI; Miller et al., 2022):

eGFR (mL/min/1.73 m^2^) = 142 × min (S_*cr*_/κ,1)^α^ × max (S_*cr*_/κ,1)^–1.2^ × 0.9938*^age^* (female × 1.012)
κ = 0.7 (female) or 0.9 (male), α = −0.241 (female) or −0.302 (male)
min(S_*cr*_/κ, 1) is the minimum of S_*cr*_/κ or 1.0
max(S_*cr*_/κ, 1) is the maximum of S_*cr*_/κ or 1.0

Proteinuria was diagnosed based on the results of proteinuria testing during a health check-up (negative, trace, + 1– + 4).

For sensitivity analyses, CKD was defined according to the ICD-10 code (N18).

### 2.4 Definition of covariates

Hypertension (HTN), diabetes mellitus (DM), hyperlipidemia, depression, body mass index (BMI), smoking, alcohol consumption, and physical activity were all considered potential confounders. The presence of HTN (I10-I13, I15), DM (E10-E14), and hyperlipidemia (E78) were defined according to the ICD-10 code with the prescription of medication within 1 year before or after the PD diagnosis, and depression (F32-F34) was defined according to the ICD-10 code. BMI, smoking status (none, ex-smoker, current, or unknown), alcohol consumption, and physical activity data were obtained from a health-screening examination database. Patient BMI was categorized according to the WHO Asia-Pacific guidelines (Weir and Jan, 2019) as underweight (< 18.5 kg/m^2^), normal (18.5– 22.9 kg/m^2^), overweight (23.0–24.9 kg/m^2^), obese (≥25.0 kg/m^2^), and unknown. Alcohol consumption was categorized as none (0 cups/week), moderate (1–14 cups/week), heavy (≥15 cups/week), or unknown. Physical activity was categorized as active (high-intensity physical activity performed ≥3 days/week or moderate-intensity physical activity performed ≥5 days/week), none (other activities), and unknown.

### 2.5 Definition of outcome and follow-up

The outcome of the study was death, which was obtained from death records of the Korean National Statistical Office. Patients were followed-up from the date of PD diagnosis (baseline) to the date of death, or until the end of the study period (December 31, 2020).

### 2.6 Statistical analysis

Independent *t*-tests and chi-square tests were used to compare the demographic and clinical characteristics of patients with PD. To show the survival curve of patients with PD and to examine the differences in the effects of CKD and proteinuria on mortality in patients with PD, Kaplan-Meier curves were plotted. To investigate the association between CKD, proteinuria, and mortality, Cox proportional hazards analyses were performed, using CKD or proteinuria as predictors after controlling for age, sex, BMI, HTN, DM, hyperlipidemia, depression, smoking, alcohol consumption, and physical activity. Additionally, interaction terms were introduced to test whether the associations between CKD and mortality differed by age (using 65 years as the cutoff) or by sex. Sensitivity analyses using the degree of eGFR and proteinuria rather than the presence of CKD and proteinuria were further performed to validate the effect of CKD and proteinuria on mortality in patients with PD. All reported *p*-values were two-sided and the significance level was set at 0.05. Additionally, to compensate for the limitation of a single measurement of eGFR at a single time point, another sensitivity analysis was performed using more stringent definition of CKD using ICD codes rather than the presence of CKD defined by eGFR. All analyses were performed using SAS version 9.4 (SAS Institute Inc., Cary, NC, USA) and R version 4.3.0 (Institute for Statistics and Mathematics, Vienna, Austria)^2^.

## 3 Results

### 3.1 Demographics and baseline characteristics

Demographic and baseline characteristics of the patients are shown in Table 1. In total, 59,293 patients with PD were enrolled in this study, of whom 9,684 had CKD (16.6%). The mean eGFR was 84.29 ± 13.71 for none-CKD group, and 47.17 ± 13.45 for the CKD group. Significant differences in baseline characteristics were observed between the non-CKD and CKD groups; patients in the CKD group were older and had a higher frequency of late-onset PD (*p* < 0.001). The CKD group also had a higher proportion of female patients and a higher BMI. Additionally, patients with CKD had more comorbidities, including HTN, DM, hyperlipidemia, coronary heart disease, ischemic stroke, osteoporosis, and depression. More patients with CKD were non-drinkers and non-smokers, and the frequency of physical inactivity was higher in the CKD group.

**TABLE 1:** Baseline characteristics.

	Overall	None CKD	CKD	*P*-value
No. participants	59,293	49,609	9,684	
Median follow up period (IQR), year	6.1	6.3	5	
Age, mean ± SD	68.6 ± 9.9	67.5 ± 9.9	74.2 ± 7.7	<0.001
Sex, female	33,462 (56.4)	27,439 (55.3)	6,023 (62.2)	<0.001
BMI, mean ± SD	23.9 ± 3.2	23.8 ± 3.2	24.1 ± 3.3	<0.001
BMI				<0.001
Underweight (<18.5)	2,417 (4.1)	2,071 (4.2)	346 (3.6)	
Normal (18.5–22.9)	20,978 (35.4)	17,751 (35.8)	3,227 (33.3)	
Overweight (23.0–24.9)	15,131 (25.5)	12,744 (25.7)	2,387 (24.7)	
Obesity (≥25.0)	20,640 (34.8)	16,939 (34.1)	3,701 (38.2)	
Unknown	127 (0.2)	104 (0.2)	23 (0.2)	
**Condition at baseline**				
Hypertension	31,476 (53.1)	24,677 (49.7)	6,799 (70.2)	<0.001
Diabetes	11,885 (20.0)	9,001 (18.1)	2,884 (29.8)	<0.001
Hyperlipidemia	13,112 (22.1)	10,819 (21.8)	2,293 (23.7)	<0.001
Depression	13,219 (22.3)	10,852 (21.9)	2,367 (24.4)	<0.001
**Smoking status**				<0.001
None	44,942 (75.8)	37,189 (75.0)	7,753 (80.1)	
Ex-smoker	8,888 (15.0)	7,601 (15.3)	1,287 (13.3)	
Current	5,373 (9.1)	4,748 (9.6)	625 (6.4)	
Unknown	90 (0.1)	71 (0.1)	19 (0.2)	
**Alcohol consumption (cups/week)**				<0.001
None	48,538 (81.9)	39,902 (80.4)	8,636 (89.2)	
Moderate	7,774 (13.1)	6,993 (14.1)	781 (8.1)	
Heavy	2,721 (4.6)	2,497 (5.0)	224 (2.3)	
Unknown	260 (0.4)	217 (0.5)	43 (0.4)	
**Physical activity**				<0.001
None	49,044 (82.7)	40,539 (81.7)	8,505 (87.8)	
Active	10,158 (17.1)	8,993 (18.1)	1,165 (12.0)	
Unknown	91 (0.2)	77 (0.2)	14 (0.2)	

### 3.2 Mortality rate in PD patients according to CKD and proteinuria

**FIGURE 2:**
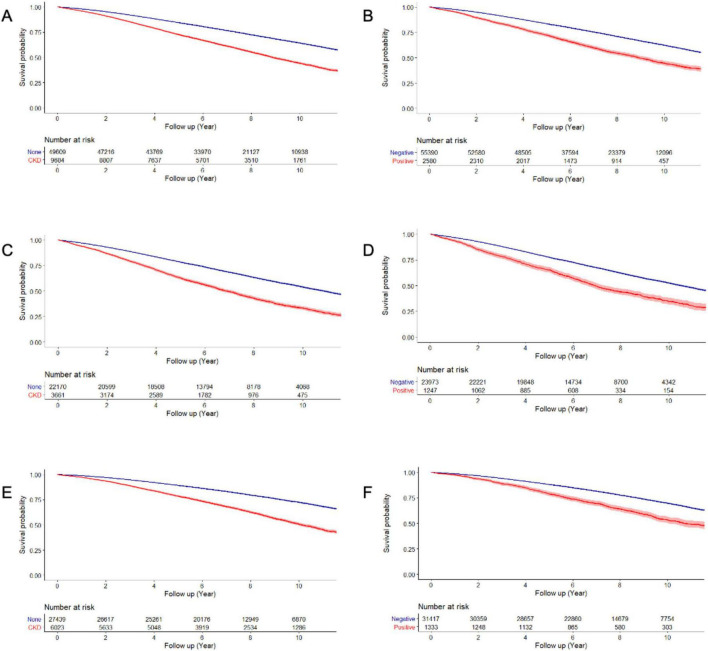
Overall survival of patients by the presence of CKD and proteinuria. Overall survival according to the presence of CKD (**A:** total, **C:** male, and **E:** female patients) and proteinuria (**B:** total, **D:** male, and **F:** female patients).

Figure 2 presents the Kaplan-Meier curves of the mortality rate in patients with PD for up to 12 years, according to the presence of CKD and proteinuria on the dipstick test. The mortality rates were 74.6 and 41.3 per 1000-person-years in the CKD and non-CKD groups, respectively; and 74.7 and 43.9 per 1000-person-years in the proteinuria and non-proteinuria groups, respectively (Tables 2, 3).

**TABLE 2:** Hazard rations (HRs) for mortality associated with CKD in PD patients.

	No. participants	No. death	Person-year	Incidence rate per 100 person-year	Adjusted HR*	*P*-value
**Total**						
None-CKD	49,609	15,266	3,69,277	4.1340	Reference	<0.001
CKD	9,684	4,867	65,220	7.4624	1.240 (1.190–1.283)	
**Male**						
None-CKD	22,170	8,810	1,54,366	5.7072	Reference	<0.001
CKD	3,661	2,214	21,792	10.1599	1.187 (1.131–1.246)	
**Female**						
None-CKD	27,439	6,456	2,14,911	3.0040	Reference	<0.001
CKD	6,023	2,653	43,429	6.1089	1.280 (1.221–1.342)	

*Adjusted for age, sex, BMI, HTN, DM, hyperlipidemia, depression, smoking, alcohol, and physical activity.

**TABLE 3:** Hazard rations (HRs) for mortality associated with proteinuria in PD patients.

	No. participants	No. death	Person-year	Incidence rate per 100 person-year	Adjusted HR*	*P*-value
**Total**						
Negative	55,390	18,001	4,09,781	4.3928	Reference	<0.001
Positive	2,580	1,277	17,100	7.4676	1.543 (1.457–1.634)	
**Male**						
Negative	23,973	9,863	1,65,621	5.9552	Reference	<0.001
Positive	1,247	725	7,393	9.8066	1.578 (1.462–1.703)	
**Female**						
Negative	31,417	8,138	2,44,160	3.3331	Reference	<0.001
Positive	1,333	552	9,708	5.6863	1.491 (1.367–1.626)	

*Adjusted for age, sex, BMI, HTN, DM, hyperlipidemia, depression, smoking, alcohol, and physical activity

Chronic kidney disease (hazard ratio [HR] = 1.240, 95% confidence interval [CI] 1.190– 1.283) was associated with higher mortality in patients with PD after controlling for potential confounders, including age, sex, BMI, hypertension, diabetes, hyperlipidemia, depression, smoking, alcohol consumption, and physical activity. We also observed significant interactions between age and sex with CKD in relation to mortality (*p* for age *x* × CKD < 0.001; *p* for sex *x* × CKD < 0.001). When applying an age cutoff of 65 years, the younger subgroup (HR = 1.746, 95% CI = 1.508–2.021) exhibited a stronger association compared with the older subgroup (HR = 1.480, 95% CI = 1.431–1.531). In addition, female patients (HR = 1.280, 95% CI = 1.221–1.342) were more vulnerable to CKD than male patients (HR = 1.187, 95% CI = 1.131–1.246) (Table 2).

Regarding the presence of proteinuria in the dipstick test, proteinuria (HR = 1.543, 95% CI 1.457–1.634) was associated with higher mortality in patients with PD. However, there was no interaction between sex and proteinuria in patients with PD (*p* = 0.603 for sex × proteinuria) (Table 3).

### 3.3 Sensitivity analyses (the degree of eGFR and proteinuria)

According to the degree of eGFR (eGFR < 30, 30–60, 60–90, ≥ 90), the HR for mortality was increased as eGFR decreased (*p* for trend < 0.001). The groups with an eGFR of 30–60 (HR = 1.193, 95% CI 1.142–1.247) and an eGFR <30 (HR = 1.845, 95% CI 1.685–2.020) were all associated with higher mortality, whereas the group with an eGFR of 60 –90 (HR = 1.004, 95% CI 0.969–1.040) was not (Table 4).

**TABLE 4:** Hazard rations (HRs) for mortality associated with eGFR in PD patients.

	No. participants	No. death	Person-year	Incidence rate per 100 person-year	Adjusted model*	*P*-value
**Total**						
≥90	19,177	4,759	145,450	3.2719	Reference	<0.001
60–90	30,432	10,507	223,827	4.6943	1.004 (0.969–1.040)	
30–60	8,731	4,339	59,076	7.3448	1.193 (1.142–1.247)	
<30	953	528	6,144	8.5932	1.845 (1.685–2.020)	
**Male**						
≥90	8,346	2,752	59,873	4.5964	Reference	<0.001
60–90	13,824	6,058	94,493	6.4110	0.966 (0.922–1.013)	
30–60	3,190	1,927	18,949	10.1697	1.104 (1.037–1.175)	
<30	471	287	2,843	10.0949	1.647 (1.457–1.862)	
**Female**						
≥90	10,831	2,007	85,577	2.3453	Reference	<0.001
60–90	16,608	4,449	129,334	3.4399	1.059 (1.003–1.117)	
30–60	5,541	2,412	40,127	6.0109	1.285 (1.206–1.368)	
<30	482	241	3,301	7.3000	2.130 (1.861–2.438)	

*Adjusted for age, sex, BMI, HTN, DM, hyperlipidemia, depression, smoking, alcohol, physical activity.

Regarding the degree of proteinuria, the HR for mortality increased as the severity of proteinuria increased (*p* for trend < 0.001). The tracer proteinuria (HR = 1.207, 95% CI 1.115–1.307), proteinuria of 1 + (HR = 1.343, 95% CI 1.245–1.448), and proteinuria ≥2 + (HR = 1.933, 95% CI 1.777–2.102) groups were associated with higher mortality (Table 5).

**TABLE 5:** Hazard rations (HRs) for mortality associated with proteinuria in PD patients.

	No. participants	No. death	Person-year	Incidence rate per 100 person-year	Adjusted model*	*P*-value
**Total**						
Negative	53,805	17,371	398,452	4.3596	Reference	<0.001
Trace	1,585	630	11,329	5.5610	1.207 (1.115–1.307)	
1+	1,563	706	10,785	6.5464	1.343 (1.245–1.448)	
≥2+	1,017	571	6,316	9.0406	1.933 (1.777–2.102)	
**Male**						
Negative	23,242	9,505	160,805	5.9109	Reference	<0.001
Trace	731	358	4,816	7.4338	1.181 (1.062–1.313)	
1+	736	398	4,542	8.7629	1.408 (1.273–1.557)	
≥2+	511	327	2,851	11.4693	1.887 (1.688–2.110)	
**Female**						
Negative	30,563	7,866	237,647	3.3100	Reference	<0.001
Trace	854	272	6,513	4.1762	1.255 (1.112–1.416)	
1+	827	308	6,243	4.9338	1.251 (1.116–1.402)	
≥2+	506	244	3,465	7.0421	2.016 (1.773–2.293)	

*Adjusted for age, sex, BMI, HTN, DM, hyperlipidemia, depression, smoking, alcohol, physical activity.

### 3.4 Sensitivity analyses using ICD codes for CKD diagnosis

We further conducted a sensitivity analysis using ICD codes for CKD diagnosis and the results were consistent with the original findings. The presence of CKD was associated with the higher mortality in patients with PD (HR = 1.645, 95% CI 1.548–1.749). The female patients showed higher HR than male patients (Table 6).

**TABLE 6:** Hazard rations (HRs) for mortality associated with CKD in PD patients.

	No. participants	No. death	Adjusted model*	*P*-value
**Total**				
None CKD	57181	18995	Reference	<0.001
CKD	2112	1138	1.645 (1.548–1.749)	
**Male**				
None CKD	24582	10271	Reference	<0.001
CKD	1249	753	1.517 (1.407–1.636)	
**Female**				
None CKD	32599	8724	Reference	<0.001
CKD	863	385	2.012 (1.816–2.230)	

*Adjusted for age, sex, BMI, HTN, DM, hyperlipidemia, depression, smoking, alcohol, physical activity.

## 4 Discussion

In this large-scale nationwide cohort-based study, we determined the impact of CKD and proteinuria on the mortality rates of patients with PD. To the best of our knowledge, this is the first study to evaluate the prognosis of PD according to the presence/absence of CKD and proteinuria. We also found that patients on PD with CKD and proteinuria had a higher risk of mortality, even after controlling for confounding factors. The degree of kidney dysfunction and proteinuria was also associated with an increased HR for mortality. Furthermore, female PD patients with CKD were found to be more vulnerable to mortality than male PD patients, whereas there was no sex-specific vulnerability of proteinuria to mortality. As such, our results underscore the importance of adopting strategies to maintain kidney function and mitigate mortality risk in patients with PD.

Our major finding, which underscores the detrimental effects of CKD on mortality in patients with PD, can be attributed to medical complications due to CKD. Previous studies have shown that CKD, defined by eGFR and proteinuria, is associated with an increased risk of all-cause mortality and cardiovascular mortality, even after adjusting for many potential confounders in the general population (Kim et al., 2019; Appel et al., 2023; Francis et al., 2024). This association is likely to present in the PD population as well, mirroring the trends observed in the general population. Alternatively, direct damages to the brain caused by CKD may explain our findings. CKD often leads to neurological complications, including encephalopathy, dementia, and Parkinsonism (Meléndez-Flores and Estrada-Bellmann, 2021), which in turn lead to increase mortality. Specifically, basal ganglia destruction due to uremia may play a role in the poor prognosis and mortality of patients with PD. Indeed, several cases have previously shown permanent damage to the basal ganglia in uremic patients with Parkinsonism, although these patients did not have PD pathology (Lee et al., 2006; Li et al., 2008). Thus, in patients with PD, uremia may aggravate basal ganglia PD pathology. In addition, CKD-related damage to cerebral small vessels may also explain our findings, given the shared damage to subcortical structures in the brain between PD and cerebral small vessel disease (CVSD). Several previous studies have shown that the association between kidney dysfunction and CSVD (Khatri et al., 2007; Wada et al., 2008; Takahashi et al., 2012). CKD might cause or coexist with cerebral small-vessel disease, which in turn leads to the deterioration of Parkinsonian symptoms. The severity of these symptoms, including frequent falls, reduced mobility, and muscle wasting, further increases the risk of mortality.

Another major finding of this study was that female PD patients with CKD were more vulnerable to mortality than male PD patients. Although the mechanisms underlying this female-specific vulnerability are not yet fully understood, this difference may be explained by sex differences in biological and socioeconomic factors. Females uniquely experience menopausal transition, which may cause or aggravate CKD-related vascular damage via decreased estrogen availability and estrogen receptor activity (Pei et al., 2017; Qian et al., 2022). Estrogen is also known to play a protective role against CKD-related microvascular diseases in premenopausal females. However, CKD may act synergistically with estrogen deficiency after menopause, leading to microvascular damage. Alternatively, prior studies have shown that estrogen may inhibit the renin-angiotensin system (RAS), thereby reducing oxidative stress and neuroinflammation (Macova et al., 2008; Labandeira-Garcia et al., 2016). In postmenopausal women with PD, an altered RAS due to CKD could exacerbate its impact on the brain, particularly as the neuroprotective role of estrogen has diminished. Beyond biological mechanisms, socioeconomic factors may also contribute to the observed sex differences in mortality among PD patients with CKD. In Korea, women often experience steeper educational and income gradients in health outcomes compared with men, and they are more likely to face economic insecurity, and have limited access to healthcare resources (Kim and Ruger, 2010). These disadvantages may further exacerbate the adverse impact of CKD on health outcomes in female patients with PD.

The impact of CKD on mortality in patients with PD also demonstrated an age-dependent pattern. The association between CKD and mortality was more pronounced in the younger subgroup, consistent with previous studies reporting a greater relative risk of death associated with CKD in younger populations. In contrast, although older patients exhibited higher absolute mortality, the relative hazard associated with CKD was attenuated, likely reflecting the influence of higher baseline mortality in advanced age (Raymond et al., 2007; Hallan et al., 2012). Taken together, these findings suggest that CKD exerts a disproportionate relative effect on mortality in younger patients with PD. From a clinical perspective, these results suggest that particular attention should be paid to identifying and managing CKD in younger patients with PD, as its relative impact on survival may be especially pronounced in this group.

The strengths of our study include the large sample size of patients with PD from the KNHIS, as well as the long follow-up period, which was sufficient to identify mortality. However, this study has some limitations. First, we lacked detailed data on the severity of PD and the specific treatments that patients received, which may have influenced the mortality outcomes. Additionally, we were unable to evaluate the effect of CKD on motor and non-motor functions of PD patients with PD. Second, we relied on a single measurement of eGFR at a single time point, which may not accurately reflect changes in kidney function over the course of the disease. This limitation could have affected the assessment of CKD severity and its association with mortality, although the use of ICD-10 diagnostic criteria helped to ensure a more reliable classification of CKD status. Third, we were unable to assess the duration of CKD, dialysis status, or specific management and treatment details related to CKD. As a result, we could not evaluate the impact of CKD management within the CKD group. Forth, our study lacked detailed information on the exact dosage and duration of antiparkinsonian medications. Levodopa or dopamine-agonist therapy has the potential to induce hypotension and exacerbate renal dysfunction (Noack et al., 2014; Stocchi et al., 2020), which may, in turn, influence mortality in the opposite direction. Consequently, the possibility of residual confounding due to uncontrolled antiparkinsonian drug exposure cannot be completely excluded. Finally, this study focused on all-cause mortality, without examining cause-specific mortality. A deeper understanding of the specific causes of death in patients with PD and CKD could provide clearer insights into how CKD contributes to mortality in this population. Despite these limitations, this study is the first to evaluate mortality in patients with PD according to their CKD status and proteinuria.

## 5 Conclusion

In conclusion, CKD and proteinuria affect all-cause mortality in patients with PD. Furthermore, CKD severity, as indicated by a lower eGFR, was found to be correlated with a higher risk of mortality. This study showed that it is important to control kidney function to prevent mortality in patients with PD. Because female patients were more susceptible to the adverse effects of CKD on mortality than their male counterparts, careful monitoring and management of kidney function would be effective in improving outcomes and mitigating mortality in female patients with PD.

## Data Availability

Publicly available datasets were analyzed in this study. This data can be found here: This study used a customized dataset from the Korean national health insurance service (KNHIS), which encompasses approximately 50 million individuals, representing more than 99% of the Korean population (http://nhiss.nhis.or.kr).

## Footnotes

1
http://nhiss.nhis.or.kr

2
https://www.r-project.org/
